# Evidences of CTLA-4 and PD-1 Blocking Agents-Induced Cardiotoxicity in Cellular and Preclinical Models

**DOI:** 10.3390/jpm10040179

**Published:** 2020-10-19

**Authors:** Vincenzo Quagliariello, Margherita Passariello, Domenica Rea, Antonio Barbieri, Martina Iovine, Annamaria Bonelli, Antonietta Caronna, Gerardo Botti, Claudia De Lorenzo, Nicola Maurea

**Affiliations:** 1 Division of Cardiology, Istituto Nazionale Tumori-IRCCS-Fondazione G. Pascale, 80131 Napoli, Italy; quagliariello.enzo@gmail.com (V.Q.); mart.iovine@gmail.com (M.I.); a.bonelli@istitutotumori.na.it (A.B.); a.caronna@istitutotumori.na.it (A.C.); 2 CEINGE—Biotecnologie Avanzate s.c.a.r.l., 80131 Naples, Italy; m.passariello@unina.it; 3 Animal Facility, Istituto Nazionale Tumori-IRCCS-Fondazione G. Pascale, 80131 Napoli, Italy; d.rea@istitutotumori.na.it (D.R.); a.barbieri@istitutotumori.na.it (A.B.); 4 Scientific Direction, Istituto Nazionale Tumori-IRCCS-Fondazione G. Pascale, 80131 Napoli, Italy; g.botti@istitutotumori.na.it; 5 Department of Molecular Medicine and Medical Biotechnology, University of Naples “Federico II”, 80131 Napoli, Italy

**Keywords:** cardiotoxicity, nivolumab, ipilimumab, immune checkpoint inhibitors, cardioncology

## Abstract

Background: Several strategies based on immune checkpoint inhibitors (ICIs) have been developed for cancer therapy, opening to advantages in cancer outcomes. However, several ICI-induced side effects have emerged in these patients, especially a rare but clinically significant cardiotoxicity with high rate of mortality. We studied the cytotoxic and pro-inflammatory properties of Ipilimumab and Nivolumab, the underlying pathways and cytokine storm involved. Methods: Co-cultures of human cardiomyocytes and lymphocytes were exposed to Ipilimumab or Nivolumab; cell viability and expression of leukotrienes, NLRP3, MyD88, and p65/NF-kB were performed. C57 mice were treated with Ipilimumab (15 mg/kg); analysis of fractional shortening, ejection fraction, radial and longitudinal strain were made before and after treatments through 2D-echocardiography. Expression of NLRP3, MyD88, p65/NF-kB, and 12 cytokines were analyzed in murine myocardium. Results: Nivolumab and Ipilimumab exert effective anticancer, but also significant cardiotoxic effects in co-cultures of lymphocytes and tumor or cardiac cells. Both ICIs increased NLRP3, MyD88, and p65/NF-kB expression compared to untreated cells, however, the most pro-inflammatory and cardiotoxic effects were seen after exposure to Ipilimumab. Mice treated with Ipilimumab showed a significant decrease in fractional shortening and radial strain with respect to untreated mice, coupled with a significant increase in myocardial expression of NLRP3, MyD88, and several interleukins. Conclusions: Nivolumab and Ipilimumab exert cytotoxic effects mediated by the NLRP3/IL-1β and MyD88 pathways, leading to pro-inflammatory cytokine storm in heart tissue.

## 1. Introduction

Immune checkpoint inhibitors (ICIs) improved overall survival in patients with cancer, opening up a new era in oncology in the last five years [1,2]. ICIs include blocking antibodies against programmed death 1 (PD-1) such as Nivolumab and Pembrolizumab [3,4]; programmed death ligand 1 (PD-L1) with Atezolizumab, Avelumab, and Durvalumab [5,6,7], or cytotoxic T-lymphocyte–associated antigen 4 (CTLA-4) inhibitors like Tremelimumab and also Ipilimumab [8,9]. However, several mechanisms are involved in immune system homeostasis as well as in cancer immune tolerance and immune-resistance, and some patients differ in PD-1 or CTLA4 expression, so different strategies are under study in clinical oncology [10]. Combinatorial ICI-based therapies (for example the association of Nivolumab and Ipilimumab) have been studied for the therapy of renal cell carcinoma, lung cancer, and melanoma; several benefits in terms of overall survival were obtained, however, at the expense of adverse cardiac events [11]. In fact, these patients reported cases of myocarditis and vasculitis [12]. Myocarditis has a prevalence of 0.06% and 2.4% in cancer patients treated with ICIs, especially when combined [12]. Myocarditis is a common immune-related adverse effect well studied in preclinical and clinical models [13], however, it was often associated with other cardiovascular events like vasculitis, venous thromboembolism, Takotsubo syndrome, atherosclerosis, and hypertension [12,14]. Despite clinical and preclinical data actually available, there is little knowledge about the biochemical mechanisms of ICI-induced cardiac toxicity and correlations with cardiac function markers such as radial or longitudinal strain and left ventricular ejection fraction are very scarce. We aimed at shedding light on some cardiac toxicity pathways associated with Ipilimumab and Nivolumab in cellular and preclinical models.

## 2. Materials and Methods

### 2.1. Cell Cultures

SK-BR-3 mammary tumor cells were cultured in Roswell Park Memorial Institute (RPMI) (1640, Gibco, Life Technologies, Paisley, UK). The human fetal cardiomyocytes (HFC) cells (Innoprot, Derio, Spain) were cultured according to the manufacturer’s recommendations, as previously described [15]. Culture media were supplemented with 10% heat-inactivated fetal bovine serum (FBS, Sigma-Aldrich, St. Louis, MO, USA), 50 U/mL penicillin, 50 μg/mL streptomycin, and 1% of l-Glutammine. All the cell lines were grown, as previously described [16].

### 2.2. In Vitro Cytotoxicity Assays

First, we tested the cytotoxic effects of Ipilimumab or Nivolumab on co-cultures of cancer cells (or HFC cells) with human lymphocytes. Cells were plated in 96-well flat-bottom plates at 10,000 cells/well for 16 h. Human peripheral blood mononuclear cells (hPBMCs) were added at the effector:target ratio 5:1 in the absence or presence of Ipilimumab or Nivolumab (200 nM), and incubated for one day under standard growth condition [17]. This dose was chosen on the basis of previous cellular experiments performed to test the antitumor and cardiotoxic effects of Ipilimumab or Nivolumab [15,16,17,18] and was lower than those reached in the patient serum/plasma administered with the conventional therapeutic doses of 10 mg/Kg. Controls included cancer cells or cardiomyocytes incubated in the absence of effector cells or in the presence of the antibodies, used alone. After the incubation with antibodies, hPBMCs were removed and adherent cancer cells or cardiomyocytes were washed twice and counted by trypan blue. Cell survival was expressed as percent of viable cells tested with Nivolumab or Ipilimumab compared to untreated cells (negative control). Cells were also imaged through a Leica Microsystems integrated microscope (DFC320).

### 2.3. Tumor Cell Lysis Assays

Tumor cell lysis was determined as previously described [19] through the quantitative analysis of Lactate dehydrogenase (LDH) using an LDH Detection Kit (Thermofisher Scientific, Meridian Rd., Rockford, IL, USA), according to the manufacturer’s instructions. Cell lysis was analyzed by measuring the fold increase of LDH in treated cells compared to that present in the supernatant of untreated cells or cells treated with an unrelated mAb, used as the negative controls.

### 2.4. Binding of Ipilimumab and Nivolumab to Mouse CTLA-4 and Lymphocytes by ELISA Assays

The Enzyme-linked immunosorbent assay (ELISA) was performed in mouse lymphocytes and recombinant protein to test the binding of antibodies anti CTLA-4, as reported in the literature [20]. Murine lymphocytes (400,000 cells/well) activated with anti-CD3/CD28 beads for two or three days were plated on round-bottom 96-well plates and incubated with antibodies in a buffer solution (PBS/Milk 2.5% volume/volume (*v/v*)) for two hours [20]. After a proper wash, plates were incubated with an anti-human IgG (H + L) HRP-conjugated antibody for 1 h. The binding of antibodies was also tested on the purified recombinant human or mouse CTLA-4/Fc or the Fc portion (as the negative control). After coating on NuncTM flat-bottom 96-well plates (5 μg/mL) and blocking with a buffer solution (PBS/milk 5% *v/v*) for 1 h, the immobilized chimeric proteins were incubated with antibodies in a buffer solution (PBS/milk 2.5% *v/v*) for two hours. After washing, plates were incubated with HRP-conjugated anti-human IgG (Fab’)2 goat monoclonal antibody in a buffer solution (PBS/milk 2.5% *v/v*) for one hour. After another washing step, plates were incubated with 3,3’,5,5’-Tetramethylbenzidine (TMB) (Sigma-Aldrich, St. Louise, MO, USA) for ten minutes, before quenching with HCl (1 M). Absorbance at 450 nm was performed through an Envision plate reader (Perkin Elmer, 2102, San Diego, CA, USA).

### 2.5. Expression of Leukotriene B4 (LTB4)

Cardiomyocytes co-cultured with human lymphocytes were treated with Ipilimumab and Nivolumab as described in the cytotoxicity test. Moreover, considering the higher incidence of cardiotoxic events in patients treated with combined Ipilimumab and Nivolumab treatment [11,12], we also measured the involvement of leukotrienes B4 in cells co-exposed to both ICIs (200 nM). After treatments, cardiomyocytes were centrifuged at 1200 rpm for 5 min and the cell pellet was used for the quantification of leukotrienes. Briefly, cell pellets were lysed in a proper lysis buffer (1 mM Ethylenediaminetetraacetic acid (EDTA), 20 mM NaF, 3 mM Na_3_VO_4_, 100 mM NaCl, 1 mM phenylmethylsulfonyl fluoride (PMSF), 50 mM Tris-HCl, and protease inhibitor cocktail). The lysates were then centrifuged, then the supernatants were collected and analyzed for quantification of leukotriene B4 (LTB4) ((5S,12R)-dihydroxy-6,14Z-8,10E-eicosatetraenoic acid) expression by using an LTB4 ELISA Kit (Enzo, Life Technology Monza, Italy), following the supplier’s instructions [21]. Data were reported as pg of leukotriene B4/mg of proteins (protein content was determined by using QuantiPro Assay, Biorad, Milan, Italy).

### 2.6. Expression of Nuclear Factor NF-Kappa-B p65 Subunit (p65-NF-kB)

Cardiomyocytes were left untreated or treated with Ipilimumab or Nivolumab, as described previously. Furthermore, in this case, we studied the NF-kappa-B p65 subunit expression in cells treated with combined Ipilimumab and Nivolumab (each at a concentration of 200 nM). After treatments, cells were harvested and lysed in a proper lysis buffer with protease inhibitor cocktail (see previous paragraph). Lysates were centrifuged and the supernatants analyzed using a TransAM NF-κB p65 Transcription Factor Assay Kit (Active Motif, Carlsbad, CA, USA), according to the manufacturer’s instructions [22]. NF-κB complexes were captured by binding to a consensus 5′-GGGACTTTCC-3′ oligonucleotide immobilized on a 96-well plate. Bound NF-κB was quantified by incubation with an anti-p65 primary antibody followed by horseradish peroxidase (HRP)-conjugated goat anti-rabbit IgG and spectrophotometric detection at a wavelength of 450 nm by using a microplate reader (xMark Microplate, Spectrofluorometer Biorad, Milan, Italy). Data were expressed as percentage of p65/NF-κB DNA binding relative to untreated cells.

### 2.7. Expression of NLRP3 Inflammasome and MyD88 

Cardiomyocytes were treated or untreated with Ipilimumab and Nivolumab, as described previously (Ipilimumab or Nivolumab or both drugs combined); after treatments, cells were harvested and lysed in a proper lysis buffer with protease inhibitor cocktail (described before). Lysates were centrifuged and supernatants were treated with an NLRP3 ELISA Kit (OKEH03368) (Aviva Systems Biology, San Diego, CA, USA) [23] or a Myeloid differentiation primary response protein MyD88 ELISA Kit (My Biosource, San Diego, CA, USA) [24] for quantification of NLRP3 and MyD88 in cells, respectively. NLRP3 expression was expressed as ng/mL; sensitivity: 0.078 ng/mL; Kit Range 0.156–10 ng/mL; MyD88 expression in cardiomyocytes was reported as pg/mL; range of lecture: 78–5000 pg/mL; and sensitivity of the assay: <46.9 pg/mL. 

### 2.8. Confocal Laser Scanning Microscope (CLSM)

Cardiomyocytes, cultured as described in the previous paragraph under standard conditions at 37 °C in a humidified 5% CO_2_ atmosphere, were seeded in a 24-well plate (5000 cells/well) and allowed to grow for 24 h. Cells were then left untreated or treated with Nivolumab or Ipilimumab or both in combination (200 nM) for 24 h. Cells were then fixed with 2.5% glutaraldehyde in PBS at room temperature for 20 min, rinsed with PBS, and permeabilized with 0.1% Triton X-100 for 5 min. The actin filaments were stained with Texas Red™-X Phalloidin—Thermo Fisher Scientific (Milan, Italy) at a concentration of 0.33 µM for 1 h; NLRP3 was stained by incubation with a primary antibody against NLRP3 (Life Span BioSciences, Seattle, WA, USA) for 1 h under gentle stirring, followed by an anti-human NLRP3 polyclonal antibody (Life Span BioSciences). The detection was performed by the addition of a Goat Anti-Rabbit IgG H & L (FITC) (ab6717, Abcam, Italy) for 1 h, under gentle stirring. Using a confocal microscope (C1 Nikon, Milan, Italy) equipped with EZ-C1 software for data acquisition and a 60x oil immersion objective, as described in the literature [25], the NLRP3 and Actin were imaged through excitation/emission at 488/515 nm and 515/620 nm, respectively.

### 2.9. Secretion of Pro-Inflammatory Cytokines

The expression of Interleukin-1β, 6, and 8 and TGF-β in cardiomyocytes were evaluated by the ELISA assay, as described in the literature [17]. Briefly, after treatments with Ipilimumab or Nivolumab, culture supernatants were centrifuged and treated for quantification of human IL-1β, 6, 8, and TGF-β ELISA kits as described in the literature [17] (Sigma Aldrich, Milan, Italy). The sensitivity of this method was below 10 (pg/mL), and the assay accurately detected cytokines in the range of 1–32,000 pg/mL.

### 2.10. Animal Models and In Vivo Studies

Sixteen C57Bl/6 mice were purchased from Harlan, San Pietro al Natisone (Italy). Mice were housed (five per cage) and maintained on a 12 h light–12 h dark cycle (lights on at 7.00 am) in a temperature-controlled room (22 ± 2 °C) with food and water ad libitum. The experimental protocols, in accordance with EU Directive 2010/63/EU for animal experiments, and Italian D.L.vo 26/2014 law, were approved by the Ministry of Health with authorization number 1467/17-PR of 13 February 2017, and institutional ethics committees: Organismo preposto al benessere degli animali (OPBA). Mice were randomized for weight in treatment groups (*n* = 8) considering an average of 20.5 g per group; the treatment groups were as follows: Control (sham), treated with intraperitoneal injection of 100 μL of water for injectable solutions, every three days until the study end point; Ipilimumab-anti-CTLA-4 (Bristol-Myers Squibb, Princeton, NJ, USA) was injected intraperitoneally at 15 mg/kg dose every three days until the study end point [26,27]. Treatments were performed for three weeks. The dose of 15 mg/kg was chosen in agreement with that conventionally used in preclinical studies aimed to analyze the cardiotoxic effects of Ipilimumab [25,26]. Furthermore, the dose of 15 mg/kg mean body-weight was also comparable to that used for clinical use of this mAb and several clinical and preclinical toxicological, anticancer, or pharmacokinetic studies (1–30 mg/kg) [28,29,30,31].

### 2.11. Echocardiographic Analysis of Ventricular Function 

To assess cardiac function in vivo, we performed non-invasive transthoracic echocardiography in sedated mice by using a Vevo 2100 high-resolution imaging system (40-MHz transducer; Visual Sonics, Toronto, ON, Canada) as reported previously [32,33]. Mice were anesthetized with tiletamine (0.09 mg/g), zolazepam (0.09 mg/g), and 0.01% atropine (0.04 mL/g). Once sedated and placed in a supine position on a temperature-controlled surgical table to maintain rectal temperature at 37 °C, continual ECG monitoring was obtained via limb electrodes. Cardiac function was evaluated by echocardiography in basal conditions and once per week for the three weeks of treatment. The LV echocardiography was assessed in parasternal long-axis views at a frame rate of 233 Hz. End-systole and end-diastole dimensions were defined as the phases corresponding to the ECG T wave, and to the R wave, respectively. M-mode LV internal dimensions, diastolic (LVID,d) and LV internal dimensions, systolic (LVID,s) were averaged from three to five beats. LVID,d and LVID,s were measured from the LV M-mode at the mid papillary muscle level. Fractional shortening percentage (%FS) was calculated as [(LVID, d-LVID, s)/LVID, d] × 100, and ejection fraction percentage (%EF) was calculated as [(EDvol − ESvol)/EDvol] × 100. The strain was measured as the deformation of the myocardial walls compared to its original size and was expressed as a percentage. The analysis started with acquired B-mode loops and were imported into the Vevo Strain software. Three consecutive cardiac cycles were selected and the endocardium was traced. Upon adequate tracing of the endocardium, an epicardial trace was added. A Segment Strain (ST) based strain allowed assessment of strains specific to six myocardial segments per LV view. Internally, 10 or plus points were measured for each of the six segments, resulting in 48 data points in total. The strain was evaluated on long-axis views as well as radial and longitudinal. Radial strain (RS), defined as the percent change in myocardial wall thickness, is reported as a positive curve reflecting increasing myocardial thickness during systole and diminishing wall thickness during diastole, representing myocardial deformation toward the center of the LV cavity. Longitudinal strain (LS) detects the percent change in length of the ventricle, typically measured from the endocardial wall in the long-axis view [32].

### 2.12. Effects of Ipilimumab Administration on Pro-Inflammatory Markers and Cytokine Profile in Heart Tissues

After treatments, mice were sacrificed after the proper anesthesia as described before. Hearts were weighed and snap-frozen in dry ice; after, heart tissues were homogenized in a solution 0.1 M PBS (pH 7.4) containing 1% Triton X-100 and protease inhibitor cocktail. Tissues were well homogenized through a step in a high intensity ultrasonic liquid processor. Obtained homogenates were than centrifuged at 4 °C and supernatants were treated for quantification of several inflammation markers. Specifically, leukotriene B4 expression (pg/mL of tissue lysate) was quantified through the LTB4 ELISA Kit (Enzo, Life Technology) [34]. Nuclear factor NF-kappa-B p65 subunit (p65-NF-Κb) expression (ng/mL of tissue extract) was quantified through a mouse, rat RelA/NF-kB p65 ELISA Kit (My BioSource, Seattle, WA, USA). NLRP3 inflammasome expression (ng/mL of tissue extract) was quantified by the NLRP3 ELISA Kit (Mouse) (OKEH05486) (Aviva Systems Biology); myeloid differentiation primary response 88 (MYD88) expression (pg/mL of tissue extract) was performed by using the mouse myeloid differentiation primary response protein MyD88 ELISA Kit (My Biosource, San Diego, CA, USA) with a detection range of 78–5000 pg/mL and a sensitivity of 46.9 pg/mL. Moreover, 12 cytokines involved in inflammation (IL-1α, IL-1β, IL-2, IL-4, IL-6, IL-10, IL-12, IL17-α, IFN-γ, TNF-α, G-CSF, and GM-CSF) were quantified in heart tissue extracts by using the 12 mouse cytokine Multiplex Assay Kit (Qiagen, Germantown, USA) following the manufacturer’s instructions. Results were expressed as pg of cytokine/mg of heart tissue [35].

### 2.13. Statistical Analyses

Data were presented as means ± standard error (SE). Analysis of variance (ANOVA) with Sidak correction for multiple comparisons was applied to compare the different groups. Values of *p* < 0.05 were considered statistically significant.

## 3. Results

### 3.1. Effects of Ipilimumab and Nivolumab on Co-Cultures of hPBMCs and Tumor or Cardiac Cells

**Figure 1:**
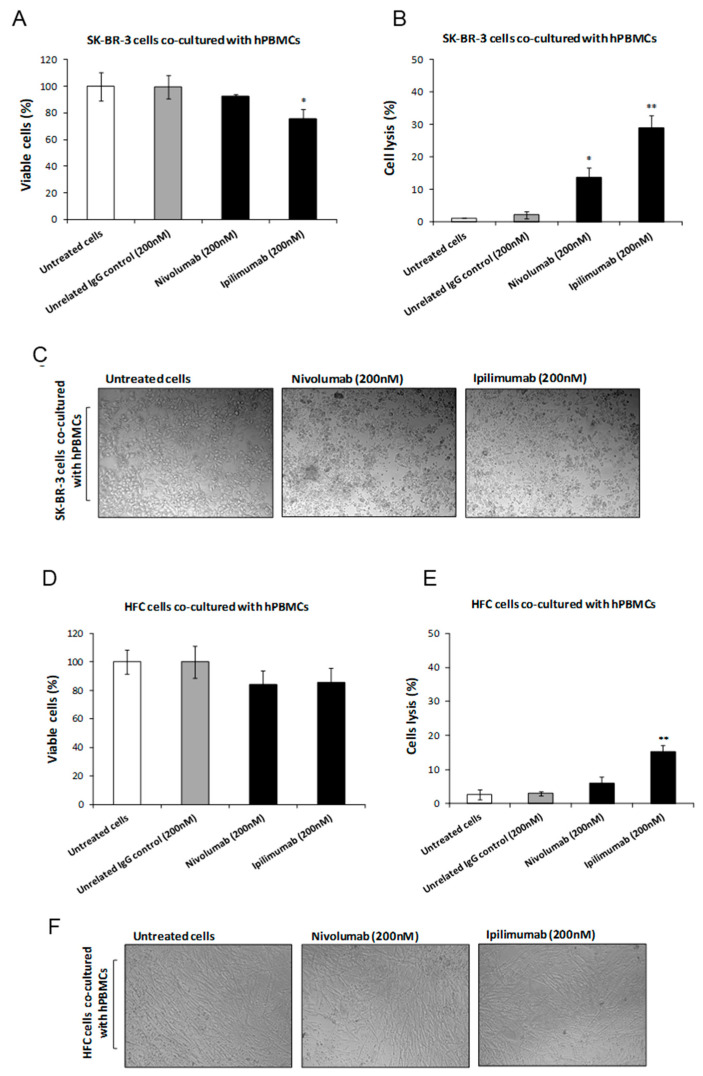
Effects of Nivolumab and Ipilimumab mAbs on SK-BR-3 tumor cells co-cultured with hPBMCs as well as on human cardiomyocytes co-cultured with hPBMCs. (**A**) Cell viability assay on SKBR-3 tumor cells, treated with Nivolumab or Ipilimumab mAb for 24 h, in the presence of hPBMCs. Untreated cells (white bars) or cells treated with an unrelated control Immunoglobulin G (IgG) (grey bars) were used as negative controls. Cells were counted by Trypan blue after the removal of lymphocytes. (**B**) LDH assay on the supernatant of co-cultures treated as indicated. Cell lysis was measured as described in the Materials and Methods section. (**C**) Representative images of SK-BR-3 tumor cells co-cultured with lymphocytes and treated as indicated. (**D**) Cell viability assay on HFC cells, treated with Nivolumab or Ipilimumab mAb for 24 h, in the presence of lymphocytes. Untreated cells (white bars) or cells treated with an unrelated control IgG (grey bars) were used as negative controls. (**E**) LDH assay on the supernatant of co-cultures treated as indicated. Cell lysis was measured as described in the Materials and Methods section. (**F**) Representative images of HFC treated in the presence of lymphocytes. Error bars depict means ± SD. *p*-values for the indicated compounds relative to untreated cells are: * *p* < 0.05; ** *p* < 0.01.

We investigated the effects of Ipilimumab and Nivolumab on co-cultures of tumor cells or human fetal cardiac (HFC) cells with human peripheral blood lymphocytes (hPBMCs), in order to compare their anti-tumor activity and eventual cardiotoxic side effects in a microenvironment similar to that of in vivo solid tumors. To this aim, tumor SK-BR-3 or HFC cells were co-cultured with hPBMCs (effector:target ratio 5:1) and treated for 24 h in the absence or in the presence of the indicated antibodies (200 nM). After the treatment, the supernatant was collected and the cells were washed to remove the lymphocytes before counting them through the trypan blue exclusion test. As shown in Figure 1A,B, both the antibodies significantly affected the viability of tumor cells, leading to increased cell lysis levels as detected by measuring the release from tumor cells of lactate dehydrogenase (LDH) in the culture medium. Ipilimumab showed a stronger effect than Nivolumab by reducing the cell viability of 20% and inducing a cell lysis increase of about 30%. Unfortunately, as shown in Figure 1D,E, similar, though milder effects of Ipilimumab and Nivolumab have been also observed on co-cultures of hPBMCs and HFC. In particular, Ipilimumab affected both the cell viability and lysis by about 15%, thus indicating that potential cardiotoxic effects are associated with its treatment, which is in line with previous results reported in the literature [36].

### 3.2. Pro-Inflammatory Markers after Treatments with Immune Checkpoint Inhibitors (ICIs)

**Figure 2:**
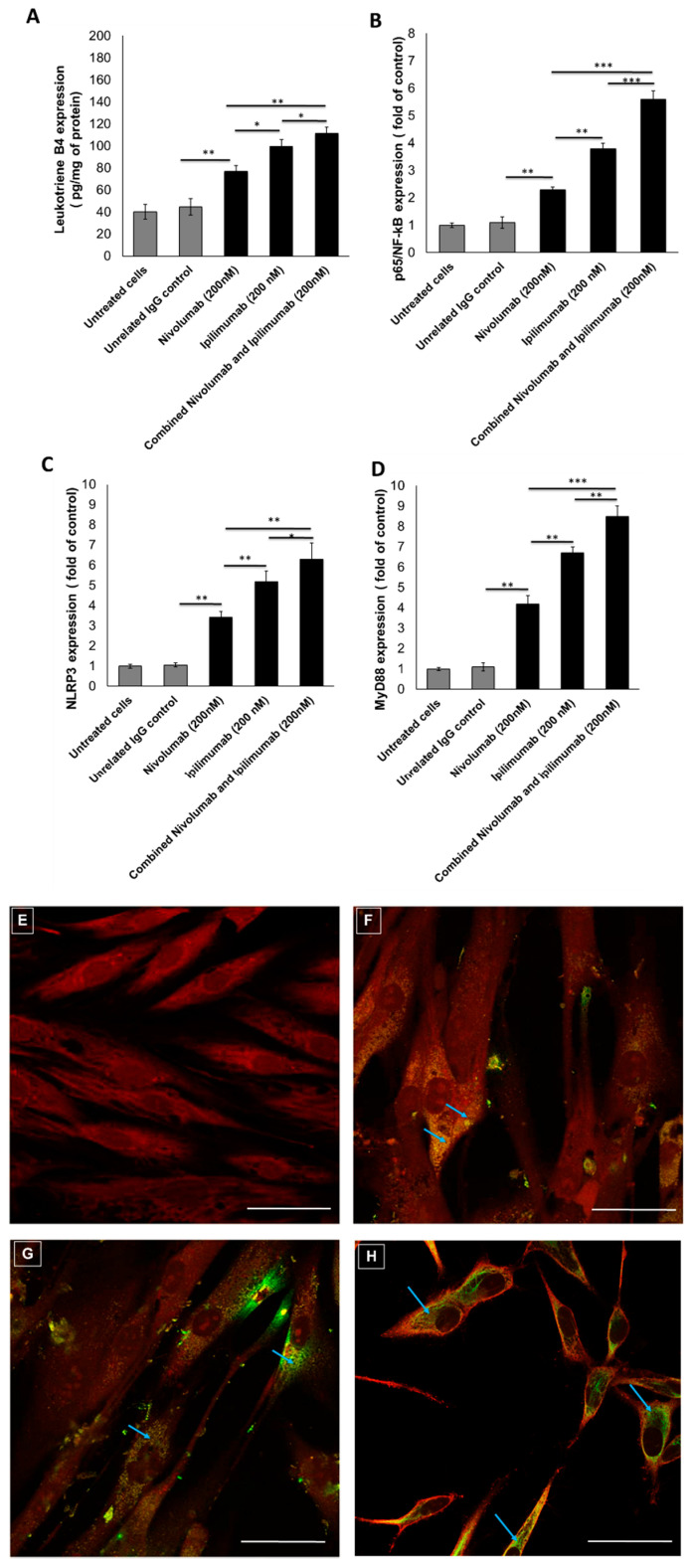
Pro-inflammatory effects of Nivolumab and Ipilimumab mAbs (alone or combined) on human cardiomyocytes co-cultured with hPBMCs. (**A**) Leukotriene type B4 production by HFC cells, treated with Nivolumab or Ipilimumab mAb alone (or combined) for 24 h, in the presence of lymphocytes. Untreated or treated cells with an unrelated control IgG (grey bars for both) were used as negative controls. (**B**) p65/NF-kB expression (fold of control) of cardiomyocytes treated as indicated before. (**C**) NLRP3 expression (fold of untreated cells) of cardiomyocytes treated with ICIs. (**D**) MyD88 expression (fold of control) of cardiomyocytes treated with ICIs. For each experiment, cell lysates were analyzed as described in the Materials and Methods section. Error bars depict means ± SD. *** *p* < 0.001; ** *p* < 0.01; * *p* < 0.05. (**E**) NLRP3 imaging through confocal laser scanning microscope (CLSM) method in cardiomyocytes unexposed or exposed to 200 nM of Nivolumab (**F**) or Ipilimumab (**G**) or Nivolumab and Ipilimumab in combination (**H**) for 24 h. Red: actin staining; Green: NLRP3 staining. Scale bar: 50 µm.

Several pro-inflammatory pathways could be related to myocardial injuries [37]. Leukotrienes are pro-inflammatory eicosanoids stimulating atherogenic prostaglandins [38]. Cardiomyocytes, co-incubated with lymphocytes exposed to Nivolumab and Ipilimumab, increased the production of leukotriene B4 (76.8 ± 5.5 pg/mL vs. 99.6 ± 6.1 pg/mL; *p* < 0.05) (Figure 2A). Compared to untreated cells, Ipilimumab and Nivolumab significantly increased the p65/NF-kB expression of 2.3 ± 0.1 and 3.8 ± 0.2 times (*p* < 0.05 for all) (Figure 2B). However, the most significant effects were seen by analyzing NLRP3 and MyD88, both involved in myocarditis and several cardiovascular diseases. Cells exposed to Nivolumab and Ipilimumab increased NLRP3 expression of 3.4 ± 0.3 and 5.2 ± 0.5 times with respect to untreated ones (*p* < 0.01 for both) (Figure 2C). Similarly, cells exposed to Nivolumab and Ipilimumab increased MyD88 expression of 4.2 ± 0.4 and 6.7 ± 0.3 times with respect to untreated ones (*p* < 0.01 for both) (Figure 2D). Moreover, the combination of Ipilimumab and Nivolumab significantly increased leukotrienes, p5/NF-Kb, NLRP3, and MyD88 compared to single treatments, thus indicating that the combination treatment of Ipilimumab and Nivolumab induced stronger pro-inflammatory effects in cardiomyocytes. The overall data clearly suggested a role of leukotrienes, NLRP3, MyD88, and p5/NF-kB in ICI-induced cardiotoxicity.

### 3.3. NLRP3 Staining by Confocal Laser Scanning Microscope

The NLRP3/Caspase 1/IL-1 pathway is a key mediator of cardiomyopathy, doxorubicin-induced cardiotoxicity, and vascular diseases [39,40]. We aimed at verifying the expression and localization of the NLRP3 inflammasome in cardiomyocytes exposed to Nivolumab and Ipilimumab through fluorescence microscopy. Unexposed cardiomyocytes have no detectable expression of NLRP3 through the CLSM method, despite the quantitative ELISA study showing a baseline expression of NLRP3 in these conditions (Figure 2F); however, after exposure to Nivolumab (Figure 5B), its expression was significantly visible in the cell cytoplasm and perinuclear region of cardiomyocytes (blue arrow). Exposure to Ipilimumab increased NLRP3 expression even more significantly (Figure 2G), as evidenced by the stronger green staining compared to the Nivolumab-treated cells (always detectable in cytoplasmic and perinuclear regions, blue arrow). These images confirm that the NLRP3 is expressed at higher levels in cells exposed to Nivolumab and Ipilimumab compared to the untreated ones. Combination treatment of Nivolumab and Ipilimumab (Figure 2H) increased the NLRP3 expression (blue arrow) in a significant manner compared to untreated cells (Figure 2E) and Nivolumab-exposed cells (Figure 2F), whereas no significant differences were observed compared to cells exposed to Ipilimumab (Figure 2G).

### 3.4. Secretion of Pro-Inflammatory Cytokines after Treatment with ICIs

**Figure 3:**
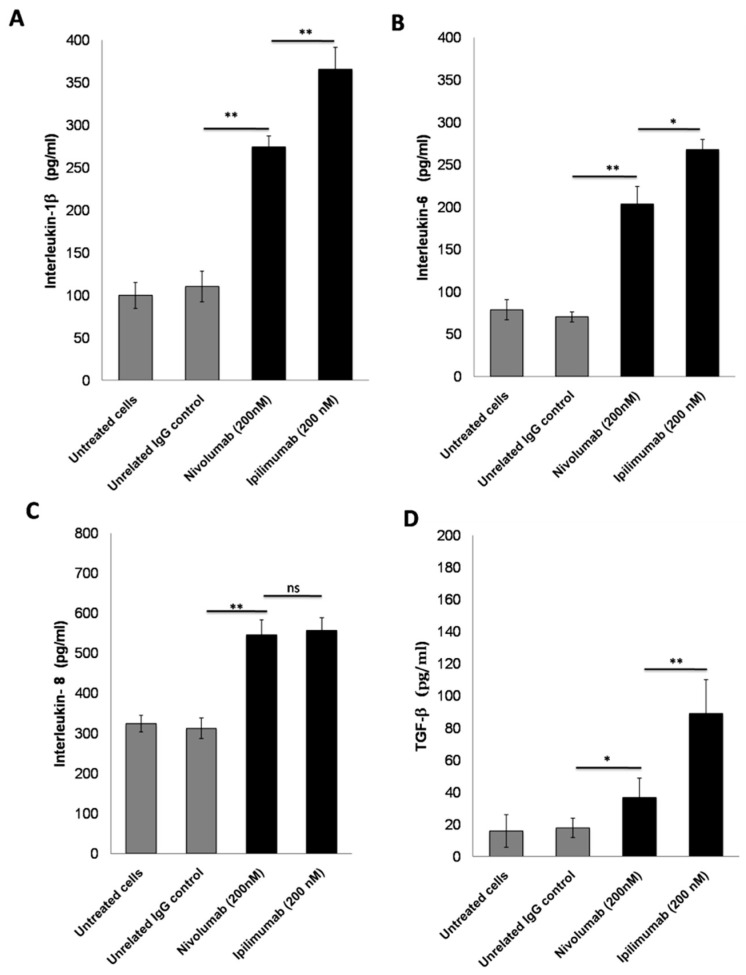
Pro-inflammatory cytokines secreted by cardiomyocytes after Nivolumab and Ipilimumab mAbs treatments. Interleukin-1 β (**A**) Interleukin-6 (**B**) Interleukin-8 (**C**), and TGF-β (**D**) production by HFC cells, treated with Nivolumab or Ipilimumab mAb for 24 h, in the presence of lymphocytes. Untreated or treated cells with an unrelated control IgG (grey bars for both) were used as negative controls. Details of the experiments are described in the Materials and Methods section. Error bars depict means ± SD. ** *p* < 0.01; * *p* < 0.05.; ns = not significant.

It is well known that some cytokines are involved in cardiovascular diseases, heart failure, and myocarditis [17,41]; however, to our knowledge, characterization of specific cytokines involved in ICI-induced cardiotoxicity have never been studied. Compared to untreated cardiomyocytes, Nivolumab significantly increased Interleukin-1 β (99.7 ± 15 vs. 274.5 ± 13 pg/mL) (Figure 3A), Interleukin-6 (78.6 ± 12 vs. 203.4 ± 21 pg/mL) (Figure 3B), Interleukin-8 (323.5 ± 21 vs. 545.5 ± 37 pg/mL) (Figure 3C), and TGF-β (15.8 ± 10 vs. 36.7 ± 12 pg/mL) (Figure 3D) (*p* < 0.01 for all). Similarly, compared to the control cells, Ipilimumab increased cellular secretion of Interleukin-1 β (99.7 ± 15 vs. 365.6 ± 26 pg/mL) (Figure 3A), Interleukin-6 (78.6 ± 12 vs. 267.8 ± 12 pg/mL) (Figure 3B), Interleukin-8 (323.5 ± 21 vs. 556.7 ± 32, pg/mL) (Figure 3C), and TGF-β (15.8 ± 10 vs. 88.9 ± 21 pg/mL) (Figure 3D) (*p* < 0.01 for all). These effects could be ascribed to the NLRP3 inflammasome and MyD88 complex, both acting as gene expression enhancers of several pro-inflammatory cytokines, chemokines, and growth factors. 

### 3.5. Preclinical Studies

#### 3.5.1. Echocardiographic Evaluation of Ventricular Function in Mice

**Figure 4:**
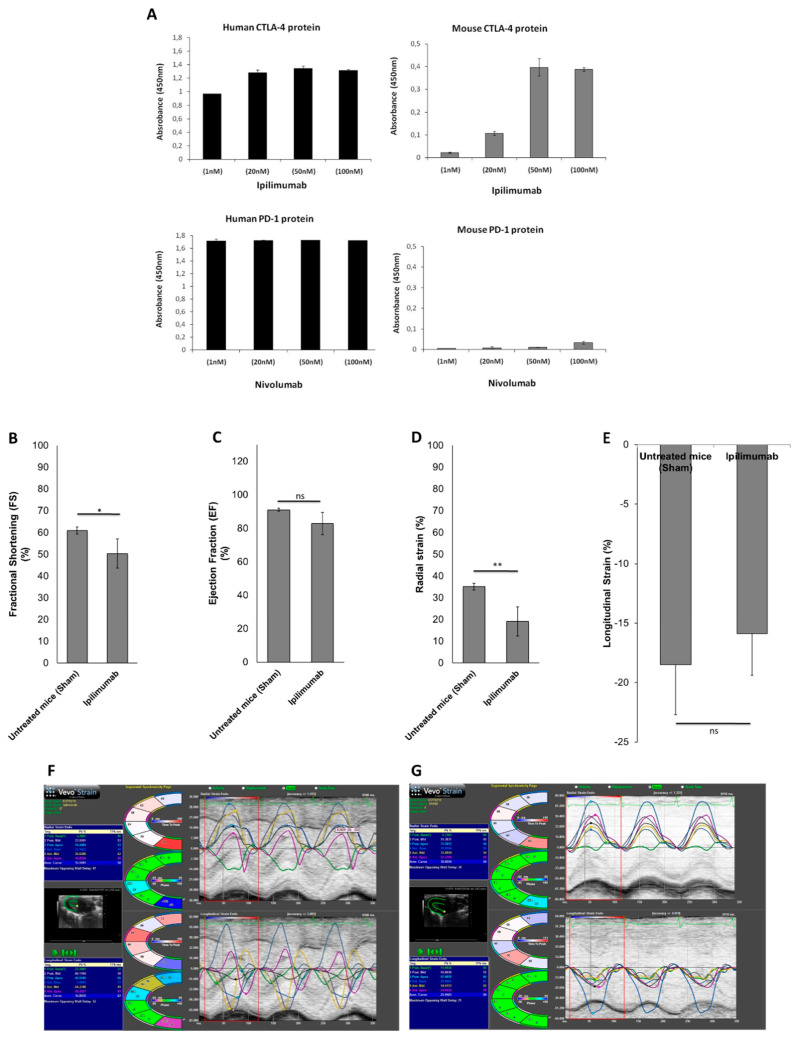
(**A**) Binding assays of Nivolumab and Ipilimumab on mouse or human purified recombinant proteins; ELISA assays of the indicated antibodies used at increasing concentrations on purified human (black bars) or mouse (grey bars) PD-1 or CTLA-4 proteins. graphic of fractional shortening (FS) (**B**) ejection fraction (EF), (**C**) radial strain (**D**), and longitudinal strain (**E**) in mice untreated (sham) or treated with Ipilimumab. Images of strain in untreated (**F**) or Ipilimumab-treated (**G**) mice. Error bars depict means ± SD. ** *p* < 0.01; * *p* < 0.05.; ns: not significant.

Cellular data demonstrated more significant cardiotoxic effects after incubation with Ipilimumab than Nivolumab. To verify the cross-reactivity of Nivolumab and Ipilimumab mAbs for the mouse target proteins, we performed ELISA binding assays by testing the antibodies at increasing concentrations on both mouse and human purified recombinant PD-1 and CTLA-4 proteins. As shown in Figure 4A, Ipilimumab binds to mouse CTLA-4 protein with a higher affinity compared to Nivolumab, which shows only a poor binding on mouse PD-1 protein. Therefore, we studied the cardiotoxic effects of Ipilimumab in mice through the evaluation of ventricular function parameters such as fractional shortening (FS), ejection fraction (EF), radial strain (RS), and longitudinal strain (LS) through 2D echocardiography (Vevo Strain 2100, Fujifilm). Ultrasound examination of the FS and EF indices showed ventricular dysfunction; in particular, the FS in the Ipilimumab group displayed a statistically significant reduction with respect to the sham: FS 50.4% ± 6.7 vs. 61.2% ± 1.6; *p* < 0.05 (Figure 4B). Strain images of Ipilimumab-treated mice (Figure 4C) showed more marked changes in both RS and LS than untreated mice, as previously described for other antibodies used in cancer therapy (e.g., pembrolizumab and trastuzumab) [17]. The RS results showed a significant decrease in the Ipilimumab group with respect to the sham: RS 19.1 ±3.8 vs. 35.1 ± 4.2.

#### 3.5.2. Pro-Inflammatory Markers in Cardiac Tissue (Leukotrienes B4, p65/NF-κB, NLRP3 Inflammasome and MyD88 Complex)

**Figure 5:**
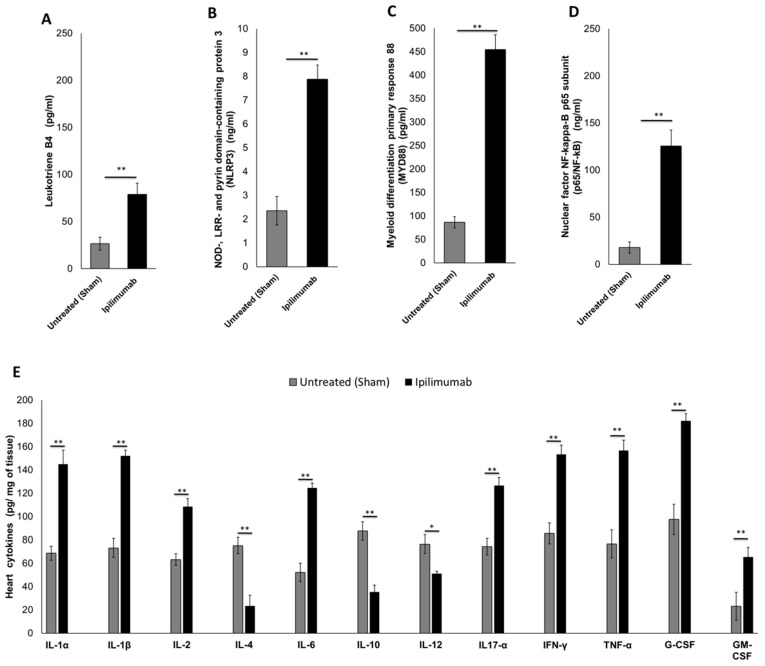
Inflammation pathways in the heart tissue of mice untreated (sham) or treated with Ipilimumab. Mice were treated as described in the Materials and Methods section; after their appropriate sacrifice, cardiac tissues were lysed and treated for quantitative analysis of leukotrienes B4 (**A**), nuclear factor NF-kappa-B p65 subunit (**B**), NLRP3 inflammasome (**C**), MYD88 (**D**), and 12 pro and anti-inflammatory cytokines (**E**). Error bars depict means ± SD. ** *p* < 0.01; * *p* < 0.05.; ns = not significant.

We investigated the cardiac markers of inflammation in mice after treatment with Ipilimumab. Compared to untreated mice, Ipilimumab increased leukotriene expression (Figure 5A) in heart lysates (78.8 ± 12.1 vs. 26.6 ± 7.2 pg/mL; *p* < 0.01) indicating the involvement of pro-inflammatory prostaglandins. The same behavior was seen in NLRP3 (7.88 ng/mL ± 0.6 vs. 2.35 ng/mL ± 0.7 for Ipilimumab and the control group, respectively; *p* < 0.01) (Figure 5B) and MyD88 expression (454.4 pg/mL ± 32.2 vs. 86.5 pg/mL ± 12 for Ipilimumab and the control group, respectively; *p* < 0.01) (Figure 5C). These proteins are NF-kB activators in cellular studies, therefore we also investigated their expression in heart lysates of mice (Figure 5D); indeed, we found the p65 subunit of NF-kB increased significantly in the Ipilimumab group with respect to the untreated mice (125.5 ng/mL ± 17.4 vs. 17.8 ng/mL ± 6.3; *p* < 0.01). These data are in line with those obtained in the cellular experiments (Figure 2), indicating the pro-inflammatory effects of ipilimumab.

#### 3.5.3. 12-Cytokines Multiplex Assay

Cytokine storm in the heart during anticancer therapies is a key driver of multiple cardiotoxic events. Therefore, we quantified multiple cytokines and growth factors in the heart tissue of Ipilimumab-treated mice (Figure 5E). First, IL-1α and IL-1β increased after Ipilimumab when compared to the untreated mice (145 ± 12 vs. 68.7 ± 5.1 pg/mg of tissue and 152 ± 6.2 vs. 73.2 ± 8 pg/mg of tissue, respectively; *p* < 0.01 compared to untreated mice for both). A similar trend was seen for IL-6 and IL17-α, both pro-inflammatory cytokines involved in myocardial damages (124.7 ± 4.2 vs. 52.2 ± 8.1 pg IL-6/mg of tissue for the Ipilimumab and control groups, respectively; 126.6 ± 7.4 vs. 74.3 ± 7.7 pg IL17-α/mg of tissue for the Ipilimumab and control groups, respectively; *p* < 0.01 for all). The only exceptions were represented by IL-4 and IL-10, which showed a decreased concentration in the heart of Ipilimumab-treated mice compared to the sham (*p* < 0.01). Moreover, INF-γ, a typical Th1 response mediator, was higher (*p* < 0.01) in the Ipilimumab group than that observed in the untreated mice. Importantly, a highly significant increase was observed in the heart levels of TNF-α, G-CSF, and GM-CSF in the Ipilimumab group compared to the control.

## 4. Discussion

Immune checkpoint inhibitors (ICIs) are antibodies against cytotoxic T lymphocyte antigen 4 (CTLA4), programmed cell death-1 (PD-1), and PD-ligand 1 (PDL-1) which have marked the history of modern oncology, being effective treatments for many types of tumors both as monotherapies and in association with radiotherapy [41,42], tyrosine kinase inhibitors [43], and standard chemotherapy [44]. However, the side effects of ICIs are a key point to consider in the clinical management of cancer patients; the most frequent adverse events are rash (maculopapular, lichenoid), pruritus, vitiligo, diarrhea, colitis, lichenoid mucositis, hypothyroidism, hyperthyroidism, thyroiditis, hypophysitis, transaminitis, hepatitis, pneumonitis, arthralgia, inflammatory arthritis, myalgia, increase in serum creatinine, nephritis, and sensorimotor neuropathy [45]. Although rare, ICI cardiotoxicity is a serious side effect that results in cardiomyopathy, myocarditis, and pericarditis [46]. A recent World Health Organization report (VigiBase) stated that ICI treatment led to an 11-fold increase in the incidence of myocarditis with respect to other treatments, with a fatality rate of 46% in combinatorial therapies compared to monotherapies [47]. Nivolumab and Ipilimumab are associated with cardiovascular injuries, especially when administered in combination and epidemiological data are variable [11]. First, Nivolumab-induced myocarditis occurs in 0.06% of patients vs. 0.27% in combination with Ipilimumab, and severe myocarditis in 10 and 60% of the cases, respectively [48]. Second, the main cardiovascular outcomes in the combination of Ipilimumab and Nivolumab are arrhythmias (0.4%), atrial fibrillation (0.2%), tachycardia (1.3%), and hypertension (1.1%) [49]. Third, some clinical cases of Ipilimumab or Nivolumab induced cardiotoxicity have been published, for instance, after four cycles of Ipilimumab therapy, a patient experienced pericarditis [50]; another case of fulminant myocarditis after one year of Ipilimumab treatment was seen [48]; a patient with lung cancer had myocardial necrosis after Nivolumab administration (dyspnea and 9% of left ventricular ejection fraction reduction) [51]. Another case of acute lymphocytic myocarditis with Nivolumab was recently seen [52] and a case of takotsubo like syndrome with apical ballooning and cardiomyopathy in a patient with melanoma treated with Ipilimumab was recently published [53]. Notably, to date, the mechanisms and key players of Nivolumab and Ipilimumab-induced myocardial injuries are not completely understood. Immune cell uptake and infiltration in myocardial tissue are always seen in human histological studies with high amounts of CD4+/CD8+ T lymphocytes and macrophages (CD68+ cells) that involve some chemokines like CXCR3, 9, and 10, which increase granzyme B-mediated cytotoxicity, driving cardiac injury [47,48,49]. However to our knowledge, the key players of Ipilimumab and Nivolumab cardiotoxicity and cytokine storm involved have never been studied in cardiac inflammation and myocardial injuries. Here, we showed for the first time that Nivolumab, and especially Ipilimumab, activates specific pathways in human cardiomyocytes. First, leukotriene B4, precursors of atherogenic, fibrogenic, and pro-inflammatory prostaglandins are always increased. The exact mechanism of leukotriene overexpression after treatment with ICIs is not totally understood, although it is conceivable that the production of reactive oxygen species due to the accumulation of lymphocytes and macrophages may increase the phospholipid oxidation with consequent activation of the arachidonate-leukotriene pathway [54]. Second, NLRP3 (inflammasome) and MyD88 (myddosome) are significantly increased after treatment with Nivolumab and Ipilimumab. Inflammasome is involved in Toll like receptor type 4 activation and is strictly involved in viral myocarditis, metabolic endotoxemia, and heart failure induced by anthracyclines. Myddosome is another complex of proteins that, through thee activation of API and NF-kB, is involved in inflammation, arthritis, severe gout, myocarditis, and heart failure. In summary, thorough direct cardiac and lymphocyte-mediated effects, Nivolumab, and especially Ipilimumab, increased pro-inflammatory cytokine storm in a myocardial microenvironment. In mice treated with Ipilimumab, a specific set of cytokines and chemokines changed: the increased cytokines were IL-1α, IL-1β, IL-6, IL-12, IL17-α, IFN-γ, TNF-α, G-CSF, and GM-CSF, indicating strong pro-inflammatory condition in myocardium after treatment with Ipilimumab. However, in contrast, IL-4 and IL-10 decreased significantly, in line with their anti-inflammatory properties; indeed IL4 inhibits the macrophage production of the IFN-γ, TNF-α, and IL-1-related pathways [55] and has been successfully used in the treatment of psoriasis [56]; IL-10 inhibits MHC class II and co-stimulatory molecule B7-1/B7-2 expression on monocytes and macrophages [57], reducing inflammation. Some considerations can be made: first, Ipilimumab seems to induce stronger cardiotoxic and pro-inflammatory effects than Nivolumab; second, NLRP3 and Myd88 are key players of Nivolumab and Ipilimumab-induced cardiotoxicity; third, their cytotoxicity was also evidenced by the significant reduction of FS and radial strain (early markers of left ventricular dysfunction [58] correlated well with increased levels of pro-inflammatory cytokines). In conclusion, cardiotoxicity is a rare adverse event induced by ICIs, but it can sometimes be fatal; we speculate that the mechanism of cardiotoxicity could be driven by NLRP3/MyD88 pathways and pro-inflammatory cytokine storm in myocardial tissue. Considering that the cardiotoxic effects observed in the animal model could be underestimated due to the reduced binding of Ipilimumab to the mouse CTLA-4 with respect to the human protein, these results highlight the urgent need of cardiac function assessment and monitoring in cancer patients following Nivolumab and Ipilimumab to prevent severe cardiac outcomes. To date, the treatment regimens for ICI-associated cardiotoxicity involves several drugs aimed to inhibit cytotoxic T cell activity against cardiomyocytes [59]. The first line treatment for myocarditis is based on steroids at high doses [60]: specifically methylprednisolone, and discontinuation of ICI in cancer patients was associated with greater improvements of myocarditis-related symptoms and MACE [61,62]. Recent consensus guidelines suggest methylprednisolone for refractory patients with ICI-induced cardiotoxicity [62]. Other proposed treatments for ICI-induced cardiotoxicity are Infliximab, a chimeric immunoglobulin G1 monoclonal antibody blocking tumor necrosis factor-α [63], anti-transplant rejection medications (e.g., anti-thymocyte globulin [ATG]) [64], CTLA-4 agonists (abatacept and belatacept) [65], tacrolimus [66], and Alemtuzumab [67], a monoclonal antibody that binds to CD52, which can result in the destruction of complement-mediated peripheral immune cells; however, despite the improvements of cardiovascular outcome, several side effects characterize their use in cancer patients (e.g., the appearance of malignant arrhythmias and severe heart failure symptoms during treatments with steroids [68], cases of heart failure in patients treated with infliximab [69], high risks of infection, and possible increases of tumor growth after treatment with CTLA-4 agonists [70]). Considering these complications, studies of new cardioprotective drugs against ICI-mediated myocarditis should be considered. Based on the pathophysiology of myocardial damage during the treatment with Ipilimumab described here, new inhibitors could be considered: first, NLRP3 or MyD88 inhibitors [71]; second, cytokine inhibitors like IL-6 blocking agents (Tocilizumab or Sarilumab, both clinically used for the treatment of rheumatoid arthritis) [72] or IL-1 or IL-1r blocking agents (e.g., Canakimumab or Anakira, both used for rheumatoid arthritis therapy and reduction of MACE in patients at high risk of heart failure) [73] or antibodies against IL-17A (Secukinumab), anti-IL-17 (Ixekizumab) and anti-IL-17 receptor A (Brodalumab) designed for the treatment of psoriasis in phase 2 trials [74]. Thus, even though the results of this study warrant further preclinical studies to fully elucidate the exact nature of these effects, it may help cardiologists and oncologists to design novel effective anti-cytokine or anti-NLRP3/MyD88 therapies against Ipilimumab-related cardiotoxicity in the near future. However, this study reveals for the first time the putative mechanisms of cardiotoxicity induced by anti PD-1 and anti CTLA-4 antibodies, but some limitations characterize it: first, we conducted a preclinical study in non-cancer bearing mice, so we did not consider eventual hormonal and cancer-related immune-modulations during treatments; second, we did not perform determination of troponins and other markers of cardiotoxicity, because in this study we aimed at finding correlations between biochemical changes and functional alterations that can predict myocardial damages in mice; and third, we carried out biological studies on monotherapy ICI without combinatorial treatments, as in the case of the association of Nivolumab and Ipilimumab (showing a stronger cardiotoxicity than its use in monotherapy). However, since Nivolumab did not show a significant binding to mouse PD-1 (Figure 4A), it was not possible to test its effects in this model, and we are currently planning further studies on humanized mouse models by using Nivolumab alone or in combination with Ipilimumab to evaluate their effects on the molecular pathways involved in multiple cardiac functions.
